# A discrete core-shell-like micro-light-emitting diode array grown on sapphire nano-membranes

**DOI:** 10.1038/s41598-020-64478-3

**Published:** 2020-05-05

**Authors:** Seungmin Lee, Jongmyeong Kim, Jehong Oh, Jungel Ryu, Kyungwook Hwang, Junsik Hwang, Sungjin Kang, Jun Hee Choi, Young Chul Sim, Yong-Hoon Cho, Tae Hoon Chung, Tak Jeong, Yongjo Park, Euijoon Yoon

**Affiliations:** 1 Department of Materials Science and Engineering, Seoul National University, Seoul, 08826 Korea; 2 Samsung Advanced Institute of Technology, Suwon, 16678 Korea; 3 Department of Physics and KI for the NanoCentury, Korea Advanced Institute of Science and Technology (KAIST), Daejeon, 34141 Korea; 4 Research Institute of Advanced Materials, Seoul National University, Seoul, 08826 Korea; 5 Inter-university Semiconductor Research Center, Seoul National University, Seoul, 08826 Korea; 6 Micro-LED Research Center, Korea Photonics Technology Institute, Gwangju, 61007 Korea

**Keywords:** Displays, Inorganic LEDs

## Abstract

A discrete core-shell-like micro-light-emitting diode (micro-LED) array was grown on a 100 nm-thick sapphire nano-membrane array without harmful plasma etching for chip singulation. Due to proper design for the sapphire nano-membrane array, an array of multi-faceted micro-LEDs with size of 4 μm × 16 μm was grown. Threading dislocation density in the micro-LED formed on sapphire nano-membrane was reduced by 59.6% due to the sapphire nano-membranes, which serve as compliant substrates, compared to GaN formed on a planar substrate. Enhancements in internal quantum efficiency by 44% and 3.3 times higher photoluminescence intensity were also observed from it. Cathodoluminescence emission at 435 nm was measured from c-plane multiple quantum wells (MQWs), whereas negligible emissions were detected from semi-polar sidewall facets. A core-shell-like MQWs were formed on all facets, hopefully lowering concentration of non-radiative surface recombination centers and reducing leakage current paths. This study provides an attractive platform for micro-LEDs by using sapphire nano-membrane.

## Introduction

Micro-light-emitting diodes (micro-LEDs) have been considered as a building block for a next-generation display technology due to their superior characteristics, compared to current technologies such as liquid-crystal display and organic light-emitting diode display^1–3^. High brightness, long lifetime, and low energy consumption are essential to outdoor and large-area displays. Especially, for the virtual and augmented reality applications, high response rates and high pixel densities are required for fast scene transitions and natural imaging without discomfort^4,5^. Because there are limitations in these requirements with the conventional display technologies, micro-LEDs draw a lot of attention as the next-generation display technology to deal with the new emerging markets^5^. Besides, micro-LEDs also exhibit enhanced light extraction, uniform current spreading, and improved heat dissipation due to their small configuration^6–9^. Therefore, since Jiang *et al*. demonstrated the first micro-LEDs with diameter of 12 μm^10,11^, researches on micro-LEDs have been widely conducted throughout academia and industry^6–9,12–23^. A micro-LED array driven by complimentary metal-oxide-semiconductor circuitry and a full color micro-LED array with active matrix operation were reported in 2009 and 2011, respectively^12,13^. In 2016, the 6.5 μm × 6.5 μm-sized blue and green micro-LED array with 2.2 × 10^7^ cd/m^2^ were demonstrated^17^.

However, there are still some issues for mass production of micro-LED displays such as low external quantum efficiency (EQE), leakage current, and costly and time-consuming transfer process onto a display backplane, etc^5^. Among them, it has been reported that the low EQEs and significant leakage current were attributed to plasma etching process, which was conventionally used for chip singulation^15,18,19,21–23^. During the plasma etching of an LED epitaxial structure, sidewalls of multiple quantum wells (MQWs) were inevitably exposed and damaged by plasma, resulting in generation of non-radiative surface recombination centers and leakage current paths. For the conventional LED chips with size over 1 mm × 1 mm, the non-radiative surface recombination and leakage current at low injection current level were not considered as severe problems. However, as the dimension of LEDs decreased, the surface to volume ratio increased, leading to the significant reduction in EQE and increase in leakage current. Although a record of EQE of 42% was reported for 10 μm × 10 μm micro-LEDs^21^, typical micro-LEDs exhibited peak EQE under 10%^15–18,20^. As an effort to solve these problems, Tian *et al*. proposed thermal curing after the plasma etching^15^. However, the thermally-cured micro-LEDs still showed EQE under 10%. Sidewall passivation methods for micro-LEDs using dielectric materials have been also reported^22^. Although improved optical or electrical characteristics were observed due to the sidewall passivation, plasma damages to the sidewalls of micro-LEDs still remains. It was reported that leakage current increased as dimension of micro-LEDs decreased, indicating incomplete suppression of sidewall damages. Therefore, if it is possible to fabricate a micro-LED array without harmful plasma etching process for chip singulation, the EQE and electrical properties would be greatly improved.

Due to the limited size and high cost of bulk GaN substrates, the commercial GaN-based LEDs are usually grown on c-plane sapphire substrates. However, the lattice mismatch and difference in thermal expansion coefficients between GaN and sapphire substrate induce high defect densities and high compressive stress in GaN layers, which causes deleterious effects on the LED performance^24^. Recently, our group demonstrated a 26 nm-thick ultra-thin sapphire nano-membrane structure, which served as a compliant substrate for the growth of less defective and less strained GaN^25^. The strain in GaN associated with the lattice mismatch between GaN and sapphire substrate could be shared with the ultra-thin sapphire nano-membrane. For the GaN grown on top surface of each membrane, the mean spacing between misfit dislocations was increased by 40% and the threading dislocation density (TDD) was reduced by 25%, compared to those of GaN grown on a conventional 430 μm-thick sapphire substrate. Significant reduction in piezoelectric field and resultant enhanced photoluminescence (PL) intensity from InGaN/GaN MQWs grown on a 100 nm-thick sapphire nano-membrane were also observed^26^.

In this study, we demonstrated a sapphire nano-membrane array for growth of a discrete micro-LED array with size of 4 μm × 16 μm without the harmful plasma etching process. Each multi-faceted micro-LED was grown on each sapphire nano-membrane without merging with adjacent ones. TDD in the micro-LEDs grown on sapphire nano-membranes was reduced by 59.6%, compared to that grown on a conventional sapphire substrate. Improved internal quantum efficiency (IQE) was also observed from the temperature-dependent PL measurement. From the cathodoluminescence (CL) measurements, two orders of magnitude stronger emission at 435 nm was observed from the c-plane than that from semi-polar sidewall facets. The InGaN/GaN MQWs were formed both on the c-plane and semi-polar facets, forming a core-shell-like, self-passivated micro-LED, hopefully minimizing the concentration of non-radiative recombination centers and leakage current paths. Sapphire nano-membrane technology is expected to provide a powerful platform for micro-LED fabrication process without plasma etching for chip singulation.

## Experimental procedure

**Figure 1:**
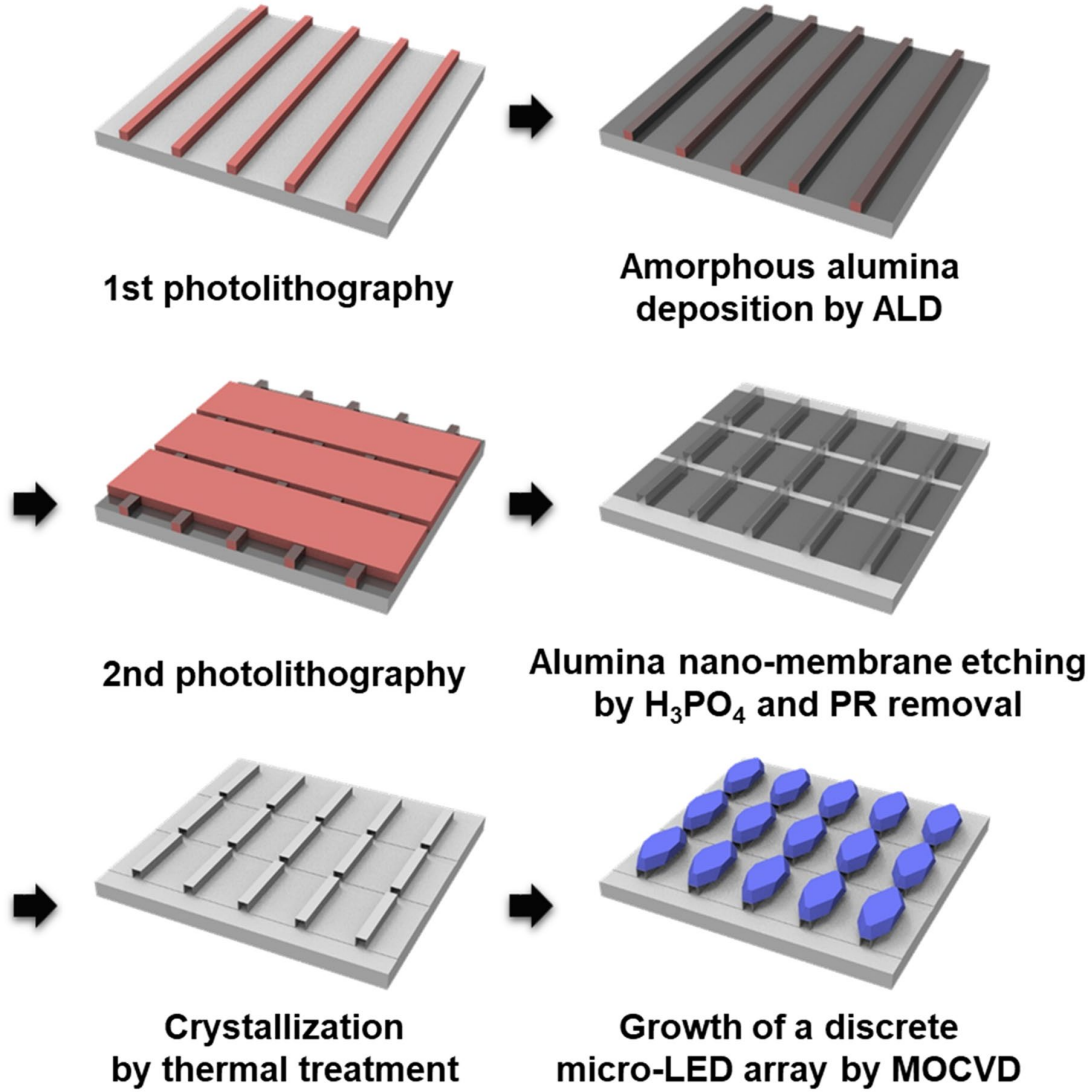
Schematic diagram for the fabrication of a sapphire nano-membrane array and the subsequent growth of a discrete micro-LED array.

The schematic sequence for the fabrication of a sapphire nano-membrane array and the subsequent growth of a discrete micro-LED array is shown in Fig. 1, and described in detail in the Method section. The sapphire nano-membrane array was fabricated by photolithography, atomic layer deposition (ALD) of amorphous alumina, alumina nano-membrane etching for the formation of an array by phosphoric acid (H_3_PO_4_), and subsequent crystallization by thermal treatment as shown in Fig. 1. During the thermal annealing, the amorphous alumina nano-membrane array was crystallized into single-crystalline sapphire (α–phase) by solid-phase epitaxy, as described in detail elsewhere^25,27^. Then, a micro-LED array could be separately grown on a sapphire nano-membrane array without chip singulation process.

## Results and Discussion

**Figure 2:**
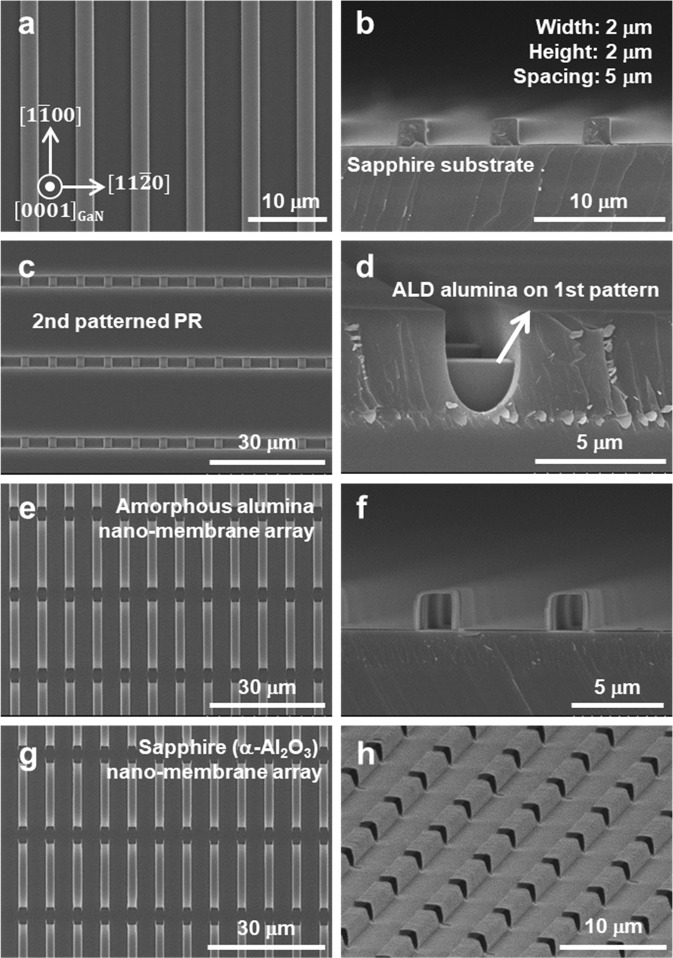
SEM images for the fabrication process of a sapphire nano-membrane array. (**a**) Plan-view and (**b**) cross-section SEM images of ALD alumina layer deposited on the first stripe-shaped PR pattern. (**c**) Plan-view and (**d**) cross-section SEM images of the second PR pattern on the ALD alumina layer. (**e**) Plan-view and (**f**) cross-section SEM images of a cavity-incorporated alumina nano-membrane array. (**g**) Plan-view and (**h**) cross-section SEM images of a sapphire nano-membrane array after thermal treatment.

Plan-view and cross-section scanning electron microscope (SEM) images of the ALD alumina-deposited first photoresist (PR) pattern are shown in Fig. 2(a,b), respectively. It was found that the stripe PR pattern was successfully formed and the amorphous alumina was conformally deposited over all exposed surface, preserving the stripe geometry. Subsequently, 4 μm-thick second PR pattern was formed in perpendicular direction with respect to the first PR pattern, exposing the top and side surface of the ALD alumina layer deposited on the first stripe PR pattern, as shown in Fig. 2(c,d). After the wet etching of exposed alumina by H_3_PO_4_, PR both above and underneath the alumina was removed, and then the cavity-incorporated amorphous alumina nano-membrane array was fabricated, as shown in Fig. 2(e,f). The width and height of the first PR pattern and the spacing between them were designed to grow a discrete micro-LED array, considering the anisotropic growth behaviors of GaN^28,29^. After the subsequent thermal treatment, the amorphous alumina nano-membrane array was crystallized into sapphire (α-phase Al_2_O_3_) nano-membrane array by solid-phase epitaxy^25,27^, as shown in Fig. 2(g,h). Due to the densification of alumina membrane during crystallization, the thickness and width of top membrane were reduced to about 100 nm and 1.7 μm, respectively. The top surfaces of the sapphire nano-membrane array were later used as templates for the growth of a discrete micro-LED array.

**Figure 3:**
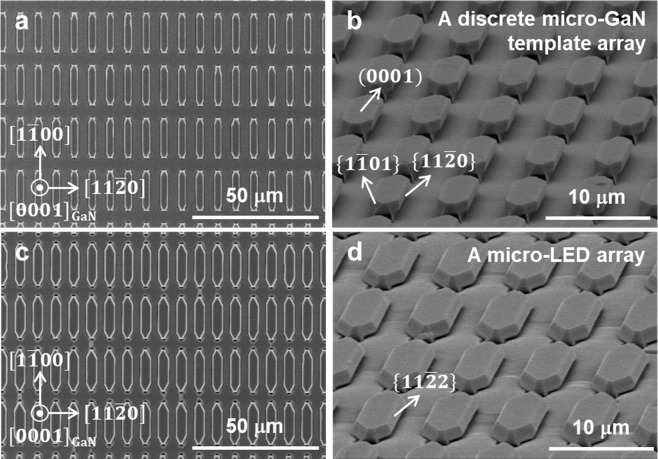
SEM images of epliayers on sapphire nano-membrane array. (**a**) Plan-view and (**b**) bird’s eye view SEM images of discrete micro-sized un-doped GaN template array. (**c**) Plan-view and (**d**) bird’s eye view SEM images of a discrete micro-LED array grown on saphire nano-membrane array.

Figure 3(a,b) show plan-view and bird’s eye-view SEM images of an array of un-doped GaN template after 40 min growth on the array of sapphire nano-membranes, respectively. An array of 3 μm-wide un-doped GaN template was obtained after the pendeo-epitaxy of GaN along the $[11\bar{2}0]$ direction of GaN. Note that $(0001)$, $\{11\bar{2}0\}$, and $\{1\bar{1}01\}$ facets were clearly observed. During the subsequent growth of an n-type GaN at a slightly different growth condition (lower temperature and higher pressure), $\{11\bar{2}2\}$ facets appeared instead of $\{11\bar{2}0\}$ facets^30^, as shown in Fig. 3(c,d). Relative growth rates of GaN facets and its configuration are greatly affected by metal-organic chemical vapor deposition (MOCVD) growth conditions^31^. Under the convex growth mode, $\{11\bar{2}2\}$ facets with slower growth rate appeared instead of $\{11\bar{2}0\}$ facets with higher growth rate during the n-GaN growth at relatively lower temperature and higher pressure^32^. The $\{11\bar{2}2\}$ facets prevailed throughout the subsequent growth of LED structures. Due to the proper design for dimensions of the sapphire nano-membrane array (*i.e*., width, length of the sapphire nano-membrane, pitches in two orthogonal directions), the micro-LEDs were isolated with respect to adjacent micro-LEDs. Note that GaN was also grown on the spacing region between the sapphire nano-membrane array; however, GaN growth was hindered since the majority of incoming Ga and N flux was more or less directed toward the GaN templates grown on the sapphire nano-membranes located at an elevated position (higher than ~2 μm with respect to the spacing region). Therefore, the precise design for the sapphire nano-membrane array is quite essential to construct a discrete micro-LED array. The shape of micro-LEDs with semi-polar $\{11\bar{2}2\}$ and $\{1\bar{1}01\}$ facets is very effective in light extraction due to reduced total internal reflection, similarly to the truncated inverted pyramid structure^33,34^. In addition to the enhanced light extraction due to nano- and micro-structures, the inclined semi-polar facets is also expected to further enhance the light extraction.

**Figure 4:**
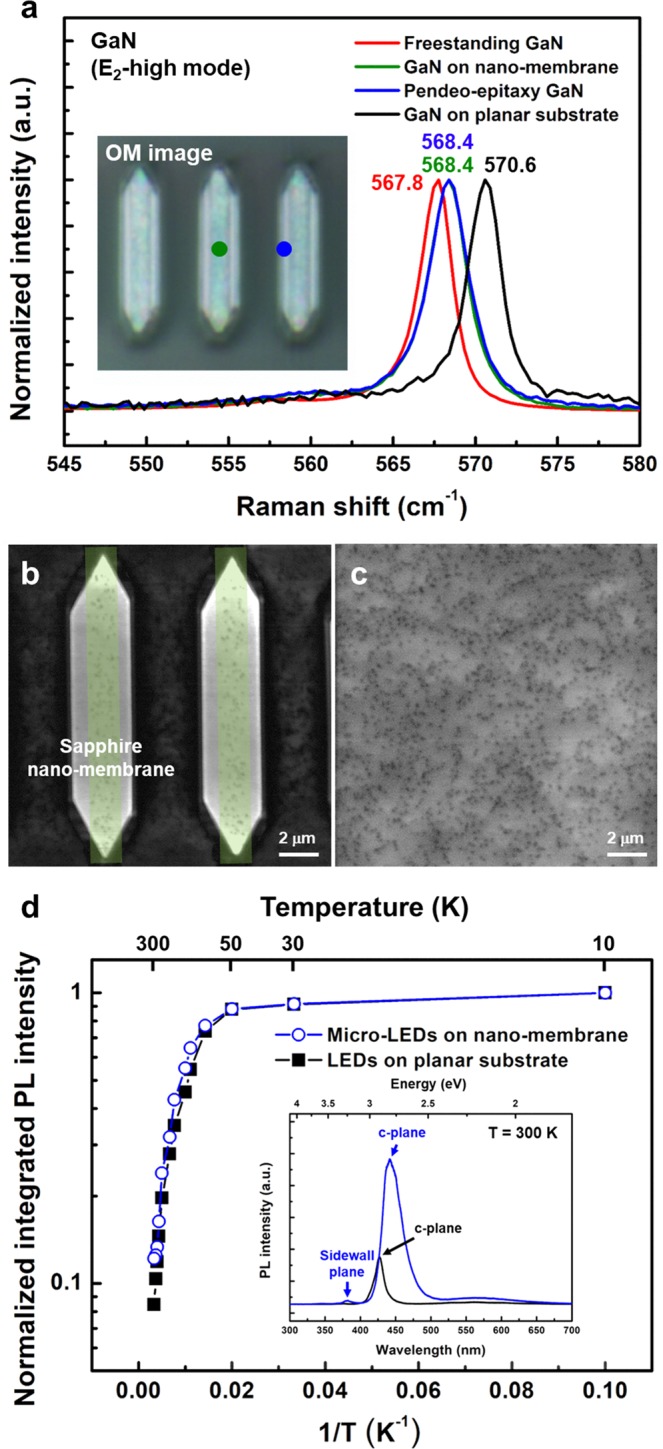
Chracterization of micro-sized un-doped GaN template and micro-LEDs (**a**) Raman spectra from freestanding GaN, micro-sized un-doped GaN on sapphire nano-membrane, and un-doped GaN on planar sapphire substrate. Panchromatic CL images of (**b**) a micro-LED grown on sapphire nano-membrane and (**c**) reference sample. (**d**) Ahrrenius plots of temperature dependent PL from micro-LEDs on sapphire nano-membrane and reference sample. The inset figure shows PL spectra measured at 300 K from micro-LEDs on sapphire nano-membrane and reference sample.

Structural and optical properties of the un-doped GaN template and the micro-LED were analyzed by micro-Raman spectroscopy, CL, and micro-PL measurements. Figure 4(a) shows Raman spectra from a free-standing GaN, an un-doped GaN template on a sapphire nano-membrane, and an un-doped GaN on a thick planar sapphire substrate. For in-depth investigation on the stress variation in the un-doped GaN on sapphire nano-membrane, Raman spectroscopy measurements were carried out at two positions, *i.e*., GaN above membrane (indicated by green dot) and pendeo-epitaxy GaN (indicated by blue dot), as shown in inset of Fig. 4(a). The peaks of E_2_-high mode were observed at 568.4 cm^−1^ from GaN at both positions, and at 570.6 cm^−1^ for GaN on planar sapphire substrate. Considering the stress coefficient of GaN (4.3 cm^-1^/GPa)^35^, the residual compressive stresses in the un-doped GaN grown on sapphire nano-membrane and planar sapphire substrate were calculated to be 139 MPa and 651 MPa, respectively. Although the extents of stress relaxation of 78.6% were slightly lower, compared to our previous results (95.7%) for ultra-thin (26 nm) sapphire nano-membrane^25^, considerable stress relaxation were observed. Figure 4(b,c) show panchromatic CL images from the micro-LEDs grown on sapphire nano-membranes and the reference sample, respectively. The locations of the sapphire nano-membranes were indicated with green boxes as shown in Fig. 4(b). TDDs from micro-LEDs in the green boxes were 2.87 × 10^8^ cm^−2^ on average, which was reduced by 37.3%, compared to that in the reference sample of 4.58 × 10^8^ cm^−2^, as shown in Fig. 4(c). Misfit dislocation is generated at the interface between epitaxial layer and substrate when the epitaxial layer thickness increases beyond so-called critical thickness. As the thickness of substrate decreases, the lattice mismatch strain in epilayer dramatically decreases due to strain partitioning. The ultra-thin nano-membrane substrate shared the part of strain in the epitaxial layer and effectively reduced the number of misfit dislocations at the GaN/nano-membrane interfaces^25^, leading to less defective GaN islands/interface and resultant reduction of TDD in the epitaxial layer^36,37^. These results are in good agreement with the stress states in GaN measured by Raman spectroscopy in Fig. 4(a). Nearly TDD-free regions were observed from the pendeo-epitaxy GaN regions due to absence of vertical propagation of threading dislocations^38^. Average TDD was measured to be 1.85 × 10^8^ cm^−2^, which was reduced by 59.6%, compared to the reference sample. Figure 4(d) shows Arrhenius plots of the integrated PL intensity from both the micro-LED on sapphire nano-membrane and the reference sample over the temperature ranges of 10 K to 300 K. The IQE was estimated from the ratio of integrated PL intensity at 10 K and 300 K by assuming that the IQE is 100% at a low temperature of 10 K^39^. The IQE of the micro-LED on sapphire nano-membrane was enhanced by a factor of 1.44, compared to the reference sample. The inset of the Fig. 4(d) shows PL spectra of the samples measured at 300 K. About 3.3 times higher integrated PL intensity was observed from the micro-LED on sapphire nano-membrane than that of the reference sample at room temperature, as shown in inset of the Fig. 4(d). The improvement in both IQE and integrated PL intensity was attributed to the stress relaxation and reduced TDD in the micro-LEDs grown on sapphire nano-membranes. The PL peak position from the micro-LEDs on sapphire nano-membranes was red-shifted by 0.12 eV, compared to that from the reference sample. The red-shift could be attributed to the higher indium incorporation into the InGaN wells^40,41^, whose temperature was slightly lower due to the lower thermal conductivity of the cavity-incorporated nano-membrane structures. Using the sapphire nano-membrane array, both blue-shift induced by reduction of quantum-confined Stark effect (QCSE) and the red-shift due to higher indium incorporation were superimposed. The apparent PL red-shift indicates that the higher indium incorporation was dominant in this study. The PL full width at half maximum (FWHM) from the micro-LEDs on sapphire nano-membranes and the reference sample were 0.17 eV and 0.10 eV, respectively. The origin of the larger FWHM will be discussed later. Additional PL peak around 382 nm fitted with the emission from the semi-polar facets as shown in CL images in Fig. 6. The laser spot size of micro PL system used in this study was 3 μm so that emission from the sidewalls could be detected from the micro PL measurements.

**Figure 5:**
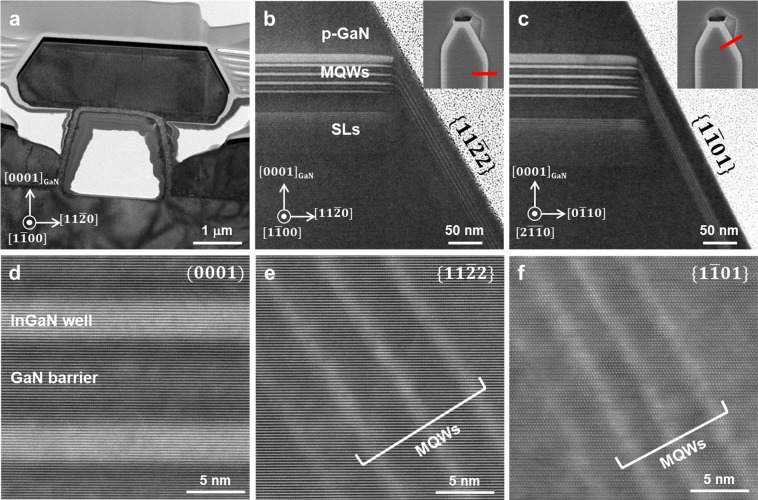
Cross-section TEM and STEM images of a core-shell-like micro-LED. (**a**) Cross-section TEM image of single micro-LED on sapphire nano-membrane with zone axis of $[1\bar{1}00]$ direction of GaN. Cross-section STEM images with zone axis of (**b**) $[1\bar{1}00]$ direction and (**c**) $[2\overline{11}0]$ direction of GaN. STEM images of InGaN/GaN MQWs on facets of (**d**) $(0001)$, (**e**) $\{11\bar{2}2\}$, and (**f**) $\{1\bar{1}01\}$.

To observe the core-shell-like MQWs on all facets of the micro-LED, transmission electron microscopy (TEM) analysis was conducted. Figure 5(a) shows a cross-section TEM image of a single micro-LED viewed along $[1\bar{1}00]$_GaN_ direction. We found that a discrete micro-LED was grown on a sapphire nano-membrane without merging with the GaN grown on the spacing region. The magnified scanning TEM (STEM) images in Fig. 5(b,c) showed that the p-type GaN layer and the InGaN/GaN MQWs were formed on all polar and semi-polar facets, implying the formation of a core-shell-like micro-LED. The red lines in insets of Fig. 5(b,c) indicate the location where cross-section images were taken. It was worth noting that thicknesses of MQWs on $(0001)$ plane near the edge gradually decreased toward both sidewalls of $\{11\bar{2}2\}$ and $\{1\bar{1}01\},$ as shown in Fig. 5(b,c). The broader PL spectrum from the micro-LEDs on sapphire nano-membranes discussed above could be explained in terms of inhomogeneous indium composition and thickness deviation in $(0001)$ facets. Funato *et al*. reported the inhomogeneous indium distributions in MQWs grown on $(0001)$ facets in the micro-structures with semi-polar and non-polar facets^38^. Cross-section STEM images of $(0001)$, $\{11\bar{2}2\}$, and $\{1\bar{1}01\}$ facets clearly show the differences in growth rate at various facets as shown in Fig. 5(d,e,f), respectively. The thicknesses of InGaN wells were measured to be 3.01 ± 0.13 nm, 1.36 ± 0.11 nm, and 1.35 ± 0.15 nm on $(0001)$, $\{11\bar{2}2\}$, and $\{1\bar{1}01\}$ facets, respectively.

**Figure 6:**
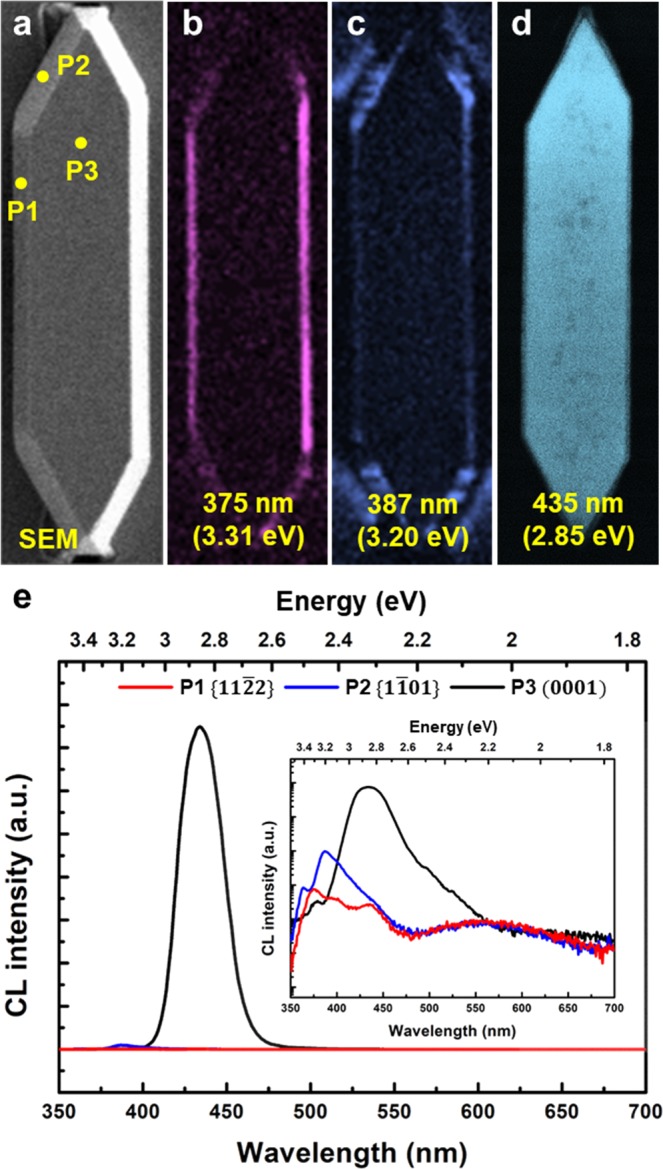
Plan-view SEM image and CL emission properties of single micro-LED (**a**) Plan-view SEM image of single micro-LED grown on sapphire nano-membrane. Monochromatic CL images measured at wavelengths of (**b**) 375 nm, (**c**) 387 nm, and (**d**) 435 nm. (**e**) Spatially-resolved CL spectra measured at $\{11\bar{2}2\}$, $\{1\bar{1}01\}$, and $(0001)$ facets. The inset figure shows the CL spectra in log scale.

Figure 6(a,b,c,d) show plan-view SEM image, monochromatic CL images at 375 nm, 387 nm, and 435 nm, respectively, from single micro-LED on a sapphire nano-membrane. The monochromatic CL measurements clarified the emission from all facets as shown in Fig. 6(b,c,d). Emissions with shorter wavelengths were measured from the facets in order of $\{11\bar{2}2\}$, $\{1\bar{1}01\}$, and $(0001)$, in good agreement with the results in the literature^42–45^. The emissions with various wavelengths were attributed to the differences in growth rates and indium incorporation on each facet^45,46^. In addition, the thinner InGaN quantum well in semi-polar facets reduced the QCSE and leads to an increased emission energy, which is consistent with the CL results. Points on each facet of the micro-LED in Fig. 6(a) indicate positions where spatially-resolved CL measurement were conducted, and the corresponding CL spectra were shown in Fig. 6(e). Peak wavelengths of the spatially-resolved CL spectra from each facet were corresponded to the monochromatic CL images shown in Fig. 6(b,c,d), respectively. Two orders of magnitude stronger emission was observed from $(0001)$ plane at 435 nm than that from semi-polar $\{11\bar{2}2\}$ and $\{1\bar{1}01\}$ planes. This result is different from emission properties of similar micro-sized light emitters with $\{11\bar{2}2\}$, $\{1\bar{1}01\}$, and $(0001)$ facets obtained from the selective growth by using a dielectic-patterned GaN/sapphire substrate^45^. Hwang *et al*. reported superior optical properties from $\{11\bar{2}2\}$ facets compared to those from $(0001)$ facet, which was explained by less QCSE at the semi-polar $\{11\bar{2}2\}$ facets. In our structures, however, the weaker CL signals from the semi-polar facets were attributed to the extremely thin InGaN wells as observed in the STEM measurements, since carriers in the very thin MQWs can be easily escape. Moreover, the ultra-thin sapphire nano-membrane significantly reduced the compressive stress in GaN, leading to reduction of QCSE in $(0001)$ facet and resultant increase in overlap of electron and hole wave functions. The strong CL emission mostly from the c-plane of the micro-LED on sapphire nano-membrane may be an important characteristic for the realization of micro-LED displays. The results suggest that the proper n- and p-metal design in micro-LEDs on sapphire nano-membranes would suppress the emission from semi-polar with shorter wavelength and generate the emission only from (0001) plane.

Development and fabrication of a vertical-type micro-LED array is currently under way. P-metal will be deposited on target wafer as well as the c-plane of micro-LED array by PR patterning and lift-off process. During the wafer bonding process, the micro-LED array can be easily transferred to the target wafer by eutectic bonding and breaking of the leg part of the sapphire nano-membrane structures. After the transfer, dielectric layers should be deposited for subsequent planarization. N-type GaN will be subsequently exposed by dry etching or chemical-mechanical polishing process. However, since plasma etching to expose n-type GaN area for n-electrode would be damaging, an alternative process such as chemical-mechanical polishing process would be needed to expose n-type GaN for n-contact formation. After the metallization for a transparent n-electrode, the vertical-type micro-LED array should be electrically operated. Comparative study of the performance of micro-LED devices on sapphire nano-membrane with those produced with plasma etching is currently under way and will be reported elsewhere.

## Conclusion

In this study, a core-shell-like micro-LED array with multi-facets was grown on a 100 nm-thick sapphire nano-membrane array. Without plasma etching for chip singulation, the core-shell-like micro-LED was individually formed on each sapphire nano-membrane. Significant stress relaxation of 78.6% in the un-doped GaN and considerable TDD reduction of 59.6% in the micro-LED were observed due to the compliant sapphire nano-membrane structure used in this study. IQE was enhanced by a factor of 1.44, and PL intensity was improved by a factor of 3.3 from the micro-LEDs on sapphire nano-membranes compared to the reference sample. Strong CL emission at 435 nm was observed from c-plane MQWs, whereas negligible CL emissions were measured from semi-polar sidewall facets. This study is expected to provide a platform technology to realize high-efficiency micro-LEDs by using sapphire nano-membrane, which is suitable for next-generation display technology.

## Method

### Fabrication of sapphire nano-membrane

A stripe-shaped PR pattern was firstly defined on a 2-inch c-plane sapphire substrate by a maskless patterning system (Nano System Solutions, DL-1000 HP) along the sapphire $[11\bar{2}0]$ direction. The width and height of the stripe were 2 μm, respectively, and the spacing between them was 5 μm. Subsequently, a 120 nm-thick amorphous alumina layer was deposited by ALD at 110 °C using trimethylaluminum and deionized water as precursors of Al and O, respectively. The second PR pattern (width: 16 μm, pitch: 20 μm) was formed on the alumina-deposited sapphire substrate along the direction perpendicular to the first stripe pattern to selectively expose the alumina layer. After the exposed amorphous alumina was etched by 85.0% H_3_PO_4_ at 50 °C, all PR was removed by acetone, resulting in a cavity-incorporated alumina membrane array. The sample was subsequently annealed at 1150 °C for 2 hours in an air ambient furnace to crystallize amorphous alumina nano-membrane into single crystalline sapphire (α–phase) by solid-phase epitaxy, as described in detail elsewhere^25,27^. The sapphire nano-membrane became epi-ready states by wet cleaning and thermal treatment with rms roughness of 0.8 nm. Through the densification of alumina membrane during the crystallization, the thickness and width of top membrane were reduced to about 100 nm and 1.7 μm, respectively.

### Epitaxial growth

An un-doped GaN array was grown for 40 mins at 1040 °C and 40 kPa in a MOCVD chamber. Micro-LED epitaxial layers including an n-type GaN layer, 15 period InGaN/GaN superlattices (SLs), an InGaN stress relaxation layer, 3 period InGaN/GaN MQWs, an InGaN quantum barrier layer^47^, and a p-type GaN layer were subsequently regrown on the un-doped GaN array in another MOCVD chamber. The n-type GaN layer was grown for 10 mins at 1030 °C and 53 kPa. Thicknesses of un-doped GaN, n-type GaN, and p-type GaN were estimated to 1.1 μm, 0.3 μm, and 0.1 μm, respectively. An array of 4 μm × 16 μm micro-LEDs were formed on the sapphire nano-membrane array after the regrowth. For comparison, a reference LED epitaxial layer was grown on a planar sapphire substrate in the same batch. However, the LED structure of the reference sample is not identical to the micro-LED sample. The quantum well thickness as well as In content in InGaN well would be slightly different due to the differences in 2-dimensional growth (reference sample) and 3-dimensional growth on sapphire nano-membrane structures. The different PL peak positions were observed, as shown in the inset of Fig. 4(d).

### Characterization

The fabrication of sapphire nano-membrane array and the growth of micro-LED array were observed by field emission scanning electron microscope, Hitachi S-4800. Raman spectra were taken from a LabRAM HV Evolution system using a 633 nm laser to analyze the stress states of un-doped GaN layers. Temperature-dependent PL were measured by Dongwoo Optron micro-PL system with a 325 nm He-Cd laser to estimate IQE. The laser spot size of micro PL system used in this study was 3 μm. Cross-section TEM images of the micro-LEDs on sapphire nano-membranes were observed by Titan G2 80–200. Cross-section STEM measurements were also conducted to observe the core-shell-like micro-LEDs at all facets. TDD (deduced from CL dark spot density), monochromatic CL images, and spatially-resolved CL spectra from each facet of micro-LEDs were analyzed by Gatan Mono-CL4 with an acceleration voltage of 5 kV at room temperature.

## Data Availability

The data that support the plots within this article and other findings of this study are available from the corresponding author upon reasonable request.
